# Population attributable fraction: planning of diseases prevention actions in Brazil

**DOI:** 10.1590/S1518-8787.2016050006269

**Published:** 2016-05-31

**Authors:** Leandro Fórnias Machado de Rezende, José Eluf

**Affiliations:** I Programa de Pós-Graduação em Medicina Preventiva. Faculdade de Medicina. Universidade de São Paulo. São Paulo, SP, Brasil; II Departamento de Medicina Preventiva. Faculdade de Medicina. Universidade de São Paulo. São Paulo, SP, Brasil

**Keywords:** Attributable Risk, Primary Prevention, Measures of Association, Exposure, Risk or Outcome, Epidemiologic Studies

## Abstract

Epidemiology is the study of occurrence, distribution and determinants of health-related events, including the application of that knowledge to the prevention and control of health problems. However, epidemiological studies, in most cases, have limited their research questions to determinants of health outcomes. Research related to the application of knowledge for prevention and control of diseases have been neglected. In this comment, we present a description of how population attributable fraction estimates can provide important elements for planning of prevention and control of diseases in Brazil.

## RESUMO

Epidemiologia é o estudo da ocorrência, distribuição e determinantes de eventos relacionados à saúde da população, incluindo a aplicação desse conhecimento para a prevenção e o controle dos problemas de saúde. Entretanto, estudos epidemiológicos, na maioria das vezes, têm limitado suas perguntas de pesquisa aos fatores determinantes de desfechos em saúde. Pesquisas relacionadas à aplicação do conhecimento para ações de prevenção e controle de doenças têm sido negligenciadas. Nesse comentário, apresentamos uma descrição de como as estimativas de fração atribuível populacional podem fornecer importantes elementos para planejamento de ações de prevenção e controle de doenças no Brasil.

## INTRODUCTION

Epidemiology is the study of the occurrence and distribution of health-related events, including its determinants, and the application of that knowledge to the prevention and control of health problems^16^. In other words, epidemiology has two central actions: 1) to identify the causes of health-related events; 2) to promote, protect and restore the health of the population^6^. One of the clearest examples of this dual action of epidemiology was evident in the fight against cholera in London. In 1849, John Snow, when mapping the cases of cholera in London, identified that the incidence of the disease was related to the distribution of water, going against the Miasma theory, prevalent at the time. In a second moment, John Snow was involved directly in actions of prevention and control of cholera, including removal of the Broad Street pump^6^ ^,^ ^20^. Recently, this transfer of knowledge from epidemiological studies to planning policies and population disease prevention actions was named “translational epidemiology”^11^.

After Snow, many other examples throughout history have managed to fill the gap between knowledge and action^5^. However, in recent history, the second action of epidemiology has been neglected. Since the beginning of the so-called “Modern Epidemiology”, epidemiologists have focused their actions, almost obsessively, in finding causal relations, using epidemiologic methods to this end. It is understandable that the field has taken such a path in the past, since little was known about the determinants of diseases in the population and, obviously, it is impossible to fight the unknown. However, even after the identification of these determinants, the same engagement with the second action related to epidemiology (application of knowledge for prevention and control of diseases) is not noticeable^6^.

The double action of epidemiology is present in the measures of association used in population studies. Two types of measure of association are used to estimate the effect of exposure in the occurrence of the disease (theoretical)^19^: 1) relative difference or incidence ratio of the disease between those exposed and not exposed to the factor; 2) absolute difference of the incidence of disease between those exposed and not exposed to the factor. The measures of association based on relative differences or ratios indicate the strength of association, and are predominantly present in etiological studies, whose main objective is to investigate the causes of health-related outcomes. Measures of association based on absolute differences, on the other hand, bring a perspective of population prevention strategies or impact on public health, since they inform the excess of risk to the disease associated with exposure. For a better understanding of the measures of association used in epidemiological studies, a vast and detailed literature is available in epidemiology textbooks^7^ ^,^ ^19^ ^,^ ^22^.

To illustrate the predominance of etiological studies in epidemiology, we performed a search on Medline on June 15, 2015, without date limits, using as descriptors measures of association based on relative (“relative differences” OR “prevalence ratio” OR “odds ratio” OR “risk ratio” OR “relative risk” OR “hazard ratio”) and absolute differences (“absolute differences” OR “excess fraction” OR “etiologic fraction” OR “impact fraction” OR “attributable fraction” OR “attributable risk” OR “population attributable risk” OR “population attributable fraction”).

In the world, we found 268,750 records based on relative differences, and only 4,784 on absolute differences. In Brazil, we found 4,352 records about the relative differences and only 82 about the absolute differences. It is worth mentioning that, possibly, part of the documents (articles, reports, books and other productions) that use measures of association based on absolute differences are in the grey literature. However, it is unlikely that the great predominance found in favor of the measures of association based on relative differences be much lower when the grey literature is considered.

In this context, we present this comment with a brief description of how estimates of population attributable fraction (PAF) can be calculated to provide important elements for planning of prevention and control of diseases actions in Brazil, enabling the approximation of epidemiologists to health care services and, consequently, to a translational epidemiology.

### Population attributable fraction estimates

PAF, based on absolute differences, estimates the proportion of the disease or health-related event that would be prevented in the population if the risk factor was eliminated. To this end, PAF provides a perspective of prevention of disease actions considering the risk of disease in exposed individuals and the prevalence of exposure in the population. Thus, high risk of disease in exposed individuals (measure based on relative difference or ratio) can have low population impact if the risk factors associated to it are rare, whereas low risk may impact public health when exposures are frequent. This concept refers to the famous Geoffrey Rose’s phrase: “A large number of people at small risk may give rise to more cases of disease than a small number of people at high risk”^18^. In this sense, as well as the other measures of association based on absolute differences, PAF provides important information about the potential impact of prevention programs and interventions in public health, being extremely useful for policymakers, managers and decision makers^19^ ^,^ ^22^.

PAF, in general, is estimated in cohort studies, in which individuals exposed and not exposed to the risk factor are followed over time, allowing to measure outcome incidence in both groups. PAF can be estimated from the following equation^13^:


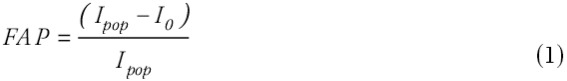


In which, *I*_pop_ is the incidence of the disease throughout the population, and *I*_o_ is the incidence of the disease in the exposed group.

However, in Brazil, cohort studies that allow the measurement of the PAF are still incipient, but there are some equations that allow to estimate it through available secondary data.

In 1953, Morton Levin showed that equation 1 is a function of the relative risk and the frequency of risk factor in the population. Therefore, PAF could also be estimated by the equation^13^:


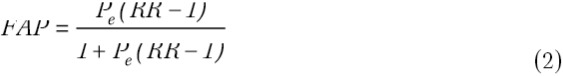


In which, *P*_e_ is the prevalence of the exposure in the population and RR is the relative risk.

Therefore, it would be possible to estimate PAF using epidemiological data on the prevalence of exposure in Brazil and the relative risk of disease to the exposure of interest. Several representative surveys of Brazilian population provide information of prevalence of risk and protective factors, allowing the stratification by the five regions, the Federation units, capitals and other cities. We can cite as examples of national surveys: *Pesquisa de Orçamento Familiar* ^9^ (Household Budget Survey), *Sistema de Vigilância de Fatores de Risco e Proteção para Doenças Crônicas por Inquérito Telefônico* ^15^ (Risk and Protective Factors Surveillance System for Chronic Diseases by Telephone Interviews), *Pesquisa Nacional de Saúde do Escolar* ^10^ (Brazilian National Survey of School Health), and *Pesquisa Nacional de Saúde* ^23^ (National Health Survey).

Relative risk, ideally, should be obtained from longitudinal studies with low risk of systematic errors and confounding. In addition, odds ratio estimates of case-control studies have also been used to estimate PAF^14^. These measures of association should be provided from studies that had the same target population for which PAF estimates would be calculated. However, often, these studies are not present in low-and middle-income countries, including Brazil. Therefore, in practice, available estimates used in systematic reviews with meta-analysis, in addition to international well-conducted cohort studies, are used. This RR portability from another target population assumes the following^21^: 1) exposure measured in the cohorts; and 2) latency of the disease are similar in the population where PAF will be estimated; 3) absence of effect modification.

The limitation inherent to equation 2 is the need to use crude RR (unadjusted). However, in most cases, associations measured in epidemiological studies are affected by confounding, and adjustment strategies are incorporated. When adjusted RR estimates are used in equation 2, a bias occurs in the measurement of PAF. In the presence of positive confounding (crude RR > adjusted RR), PAF will be underestimated, while for negative confounding (crude RR< adjusted RR), it will be overestimated. The PAF bias will depend on the magnitude of the confounding (the higher the magnitude, the greater the bias), the prevalence of confounding variable (the lower prevalence, the greater the bias), and the strength of association between exposure and outcome (the lower the magnitude, the greater the bias)^3^.

In the presence of confounding, the use of the following equation is recommended^17^:


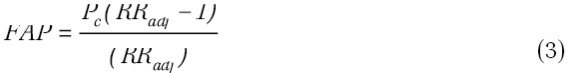


In which, *P*_c_ is the prevalence of exposure among the cases of the disease and *RR*_adj_ is the relative risk adjusted by confounding variables.

Thus, to obtain *P*_c_, it is necessary to measure the prevalence of the risk factor in population subgroups or, at least, consider the possible variation of exposure in this subgroup. A way to obtain it is to measure the prevalence ratio of exposure among the case population and the general population, reported in cohort studies. Knowing the relative difference of the prevalence of exposure among cases of the disease and the general population, this correction factor can be applied on the prevalence of exposure in the general population, to obtain the prevalence of exposure between cases of the disease. For example, the prevalence of physical inactivity among cases of colon cancer is 1.22 times that of the general population^12^. Thus, if the prevalence of physical inactivity in Brazil is approximately 20.0%, we can estimate that the prevalence of physical inactivity among the cases of colon cancer in Brazil is 24.4%.

When the exposure data and the values of relative risk are available, respectively, in a continuous way (example: gram/day) and per unit of exposure increase (dose-response RR), the following formula is used to obtain PAF:


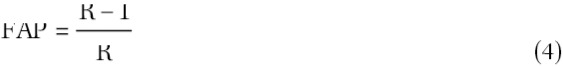


In which, R = exp [In(RR_dose_) × 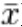]

RR_dose_ = relative risk of the disease per unit of exposure increase.


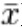
 = average of the target population’s exposure.

This equation assumes a log-linear relationship between exposure and outcome^2^.

These measures of impact in public health have been used by various organizations around the world to prioritize assistance, determine goals and start public policies. As an example, we can mention the World Health Organization, which, using the Global Burden of Disease study, provides information on the main modifiable risk factors for diseases and harms, used for the determination of targets for the reduction of non-communicable diseases by 2025^25^. In America, since 2009, the Pan American Health Organization, from the publication of estimates of deaths attributable to sodium consumption^1^, supports a Technical Advisory Group that aims to mobilize policies and interventions to reduce salt intake. In the United States, the Centers for Disease Control and Prevention (CDC) provide information of lost years of life, lost years of life adjusted by disability, and economic costs attributable to some risk factors (e.g.,smoking)^b^.

To illustrate the use of estimates in Brazil, we estimate the PAF of cardiovascular disease mortality associated with low intake of vegetables. According to data from the *Pesquisa de Orçamento Familiar* carried out in 2008, the average availability of vegetables in Brazilian homes was 74.2 grams per capita per day^9^. The minimum recommendation of intake of fruits and vegetables is 400 grams/day, which can be distributed in 240 grams of vegetables and 160 grams of fruits. A recent meta-analysis found inverse association between vegetables consumption and cardiovascular disease mortality (HR 0.96 to each serving of vegetables per day, equivalent to 77 grams/day)^24^. Using equation 4, we estimate that approximately 9.6% of deaths from cardiovascular diseases would be prevented if the minimum recommendation of daily ingestion of vegetables was reached in Brazil.

For greater understanding about PAF estimates, a vast literature can be consulted to obtain more details on the different equations, limitations, strengths and perspectives of the method^1^ ^,^ ^3^ ^,^ ^4^ ^,^ ^8^ ^,^ ^17^ ^,^ ^21^. From this, it is possible to understand some public health measures of impact derived from PAF, such as lost years of life, years of life lived with disability, and lost years of life adjusted by disability^21^. It is also possible to obtain subsidies to estimate PAF confidence intervals, by calculating the variance of PAF or even by Monte Carlo simulations^21^.

## CONCLUSION

The aim of this comment was to discuss the possibilities of use of PAF estimates by epidemiologists in Brazil and its importance for planning policies and prevention of diseases. We believe that epidemiologists have a key role in these actions, often neglected, and that, using the available secondary data, PAF estimates constitute an important means of approximation and work among epidemiologists and the health services.

## Footnotes

b Center for Disease Control and Prevention. Smoking & Tobacco Use. [cited 2015 Jun 15]. Available from: http://www.cdc.gov/tobacco/data_statistics/index.htm
